# RAREMETAL: fast and powerful meta-analysis for rare variants

**DOI:** 10.1093/bioinformatics/btu367

**Published:** 2014-06-03

**Authors:** Shuang Feng, Dajiang Liu, Xiaowei Zhan, Mary Kate Wing, Gonçalo R. Abecasis

**Affiliations:** Department of Biostatistics, University of Michigan School of Public Health, Ann Arbor, MI 48109, USA

## Abstract

**Summary:** RAREMETAL is a computationally efficient tool for meta-analysis of rare variants genotyped using sequencing or arrays. RAREMETAL facilitates analyses of individual studies, accommodates a variety of input file formats, handles related and unrelated individuals, executes both single variant and burden tests and performs conditional association analyses.

**Availability and implementation:** http://genome.sph.umich.edu/wiki/RAREMETAL for executables, source code, documentation and tutorial.

**Contact:** sfengsph@umich.edu or goncalo@umich.edu

## 1 INTRODUCTION

In genomewide association studies, meta-analysis has been key in establishing association between common variants and complex traits (Willer *et al.*, 2010). Recent advances in exome sequencing and the development of exome genotyping arrays are enabling complex disease studies to explore association between rare variants of clear functional consequence and complex traits. For these rare variants, single variant tests can lack power, and association tests that group rare variants by gene or functional unit are favored (Li and Leal, 2008; Lin and Tang, 2011; Madsen and Browning, 2009; Price *et al.*, 2010; Wu *et al.*, 2011).

Here, we describe a tool for meta-analysis of rare variant association studies for quantitative traits. Our tool enables individual studies to account for study-specific covariates as well as family and population structure. In addition, it generates summaries of linkage disequilibrium information that allow association tests for groups of rare variants during meta-analysis.

## 2 METHODS

The key idea in our implementation is that gene-level test statistics can be reconstructed from single variant score statistics and that, when the linkage disequilibrium relationships between variants are known, the distribution of gene-level statistics can be derived to evaluate significance.

Several other tools to support rare variant meta-analysis are now available (Lee *et al.*, 2013; Lumley *et al.*, 2012; Tang and Lin, 2013; Voorman *et al.*, 2013). We have tried to complement these tools by adding support for modeling of related individuals and the X chromosome, additional QC statistics, directly using compressed files to facilitate sharing and implementing conditional analyses to disentangle the contributions of nearby variants, common or rare.

RAREMETAL works in two steps. The first step, implemented in RAREMETALWORKER (RMW), analyzes individual studies and generates summary statistics that can later be combined across studies. This step can account for relatedness among individuals or hidden population structure using a variance component approach, based on either a kinship matrix estimated from pedigree (Abecasis *et al.*, 2002) or a genomic relationship matrix estimated from marker data (Kang *et al.*, 2010; Lippert *et al.*, 2011). When chromosome X is analyzed, an additional variance component is used to describe kinship for X-linked markers.

RMW tabulates single variant score statistics, which summarize evidence for association, together with covariance matrices, which summarize linkage disequilibrium relationships among variants (see our online documentation for methods http://genome.sph.umich.edu/wiki/RAREMETALWORKER_METHOD). RMW also tabulates quality control statistics for traits and covariates (mean, standard deviation and number of phenotyped samples) and marker genotypes (Hardy–Weinberg Equilibrium *P*-values and genotype missing rate). These can be used to identify problematic markers and studies during meta-analysis.

Meta-analysis is implemented in a separate tool, RAREMETAL, which calculates gene-level burden tests (either weighted or unweighed), variable frequency threshold tests and sequence kernel association tests (SKAT) (Liu *et al.*, 2014). Key formulae can be found in our online documentation (http://genome.sph.umich.edu/wiki/RAREMETAL_METHOD).

RAREMETAL can also use variance–covariance matrices to perform conditional analyses that distinguish true signals from the shadows of significant variants nearby.

## 3 RESULTS

One of our primary considerations in RAREMETAL was support for standard, easy-to-implement input formats. RMW uses Merlin format input files (Abecasis *et al.*, 2002) to retrieve phenotypes, covariates and family structure and VCF files to retrieve genotypes (Danecek *et al.*, 2011). Checks are implemented for a variety of problems in input files, including formatting errors, X-linked genotypes that are inconsistent with reported sex and matching of identifiers across files.

RMW and RAREMETAL are implemented in C++. Source code and binary executables are available from our web site. For convenience, input and output files can be processed directly in GZIP format. We have also tested compilation on several Linux, MAC OS X and Windows platforms.

### 3.1 Usage

RMW and RAREMETAL runs can be customized through command line parameters. These allow users to specify whether phenotypes should be quantile normalized, whether covariates should be modeled, whether population and/or family structure should be controlled using variance components, the size of linkage disequilibrium matrices to be shared (customized through a window size parameter) and boundaries between pseudo-autosomal and sex-linked regions of the X chromosome.

**Fig. 1.**
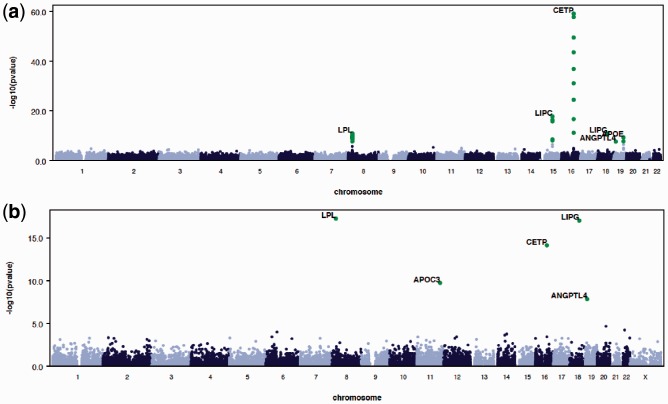
Automatically generated QQ and Manhattan plots by RAREMETAL and RMW. (**a**) Manhattan plot from single variant analysis. (**b**) Manhattan plot from gene-level burden tests

A unique feature of RAREMETAL is the ability to customize variant groupings for gene-level statistics at the meta-analysis stage, after individual studies are analyzed. RAREMETAL generates separate reports for each gene-level test with detailed information. QQ and Manhattan plots can be generated by RMW and RAREMETAL directly (see Fig. 1 for example).

RAREMETAL is already being used in large meta-analyses of rare variants for a variety of traits, ranging from blood lipids levels, anthropometric traits to smoking and drinking.

### 3.2 Performance

Using RMW, generating per study statistics in a recent analysis of exome array genotypes at 238 000 markers in 2000 individuals required between ∼9.1 min (unrelated samples) and ∼26.8 min (using genomic relationship). Using RAREMETAL, meta-analysis of 23 studies (sample size of ∼51 000) required ∼40 min to produce single variant and all available gene-level association test results across ∼18 000 genes.

### 3.3 Comparison to other tools

When analyzing ∼6000 unrelated individuals at ∼100 000 markers, RMW/RAREMETAL provides a speed improvement of ∼600-fold compared with SCORESEQ/MASS (Tang and Lin, 2013). This difference in speed increases with sample size and number of studies. The R package metaSKAT (Lee *et al.*, 2013) provides comparably fast computations, but variable threshold test is not provided. An important difference between RAREMETAL and these published tools is the ability to use linear mixed models to account for sample relatedness and/or population structure. Even when using linear mixed models to account for relatedness and population structure, RMW can handle large datasets. A mixed model analysis of 10 000 individuals at 238 000 markers used 6.1 h and 2 GB memory. With 12 GB memory, RMW was able to analyze 23 000 individuals in <5 days. Other features in RAREMETAL unique to other published tools are the flexibility of changing gene definitions and grouping strategies after individual studies have been analyzed and the ability to perform conditional meta-analysis.

In contrast to popular single variant meta-analysis methods, such as implemented in METAL (Willer *et al.*, 2010), our new approach is expected to provide more power for analysis of rare variants (Liu *et al.*, 2014). We hope RAREMETAL will accelerate the discovery of trait-associated rare variants, leading to insights into human biology.
